# Testing the effectiveness of REACH Pregnancy Circles group antenatal care: protocol for a randomised controlled pilot trial

**DOI:** 10.1186/s40814-018-0361-x

**Published:** 2018-11-10

**Authors:** Meg Wiggins, Mary Sawtell, Octavia Wiseman, Christine McCourt, Lauren Greenberg, Rachael Hunter, Sandra Eldridge, Penny Haora, Inderjeet Kaur, Angela Harden

**Affiliations:** 1 University College London Institute of Education, London, UK; 2 City, University of London, London, UK; 3 University of East London, London, UK; 4 Queen Mary University, London, UK; 5 Barts Health NHS Trust, London, UK; 6 University College London, London, UK

**Keywords:** Maternity care, Group antenatal care, Pilot trial, Ethnic diversity, Progression criteria

## Abstract

**Background:**

Antenatal care is an important public health priority. Women from socially disadvantaged, and culturally and linguistically diverse groups often have difficulties with accessing antenatal care and report more negative experiences with care. Although group antenatal care has been shown in some settings to be effective for improving women’s experiences of care and for improving other maternal as well as newborn health outcomes, these outcomes have not been rigorously assessed in the UK. A pilot trial will be conducted to determine the feasibility of, and optimum methods for, testing the effectiveness of group antenatal care in an NHS setting serving populations with high levels of social deprivation and cultural, linguistic and ethnic diversity. Outcomes will inform the protocol for a future full trial.

**Methods:**

This protocol outlines an individual-level randomised controlled external pilot trial with integrated process and economic evaluations. The two trial arms will be group care and standard antenatal care. The trial will involve the recruitment of 72 pregnant women across three maternity services within one large NHS Acute Trust. Baseline, outcomes and economic data will be collected via questionnaires completed by the participants at three time points, with the final scheduled for 4 months postnatal. Routine maternity service data will also be collected for outcomes assessment and economic evaluation purposes. Stakeholder interviews will provide insights into the acceptability of research and intervention processes, including the use of interpreters to support women who do not speak English. Pre-agreed criteria have been selected to guide the decision about whether or not to progress to a full trial.

**Discussion:**

This pilot trial will determine if it is appropriate to proceed to a full trial of group antenatal care in this setting. If progression is supported, the pilot will provide authoritative high-quality evidence to inform the design and conduct of a trial in this important area that holds significant potential to influence maternity care, outcomes and experience.

**Trial registration:**

ISRCTN ISRCTN66925258. Registered 03 April 2017. Retrospectively registered.

**Electronic supplementary material:**

The online version of this article (10.1186/s40814-018-0361-x) contains supplementary material, which is available to authorized users.

## Background

Antenatal care is an important public health priority as it has the potential to impact positively on women’s health during pregnancy and upon the subsequent life course of women and their children. Women from socially disadvantaged and ethnic minority groups often have difficulties with accessing antenatal care [1] and report more negative experiences with care, despite having potentially complex social and medical needs [2]. Lack of engagement with antenatal care has been associated with adverse pregnancy outcomes including low birthweight, neonatal mortality and maternal mortality [3–5].

Group antenatal care combines conventional aspects of antenatal assessment with information sharing, including group discussion and learning, and the opportunity for social support for pregnant women. It is facilitated by health professionals (often midwives) for small groups of women with similar estimated due dates (and potentially their partners). To date, group-based models have been successfully implemented in a number of countries worldwide, including Australia [6], Sweden [7] and the USA [8]. Antenatal care configured in this way has been shown to increase women’s satisfaction with care and has improved health and safety outcomes such as pre-term birth and low birthweight [8–10].

Antenatal care for women in groups addresses multiple factors that have been found to be associated with women’s negative experiences of antenatal care [11–14]. As each appointment lasts for approximately 2 h (compared with approximately 15–20 min for a standard antenatal care appointment) and is facilitated by the same health professionals at each session, this model increases the amount of time that a pregnant woman spends with caregivers, e.g. midwives [6, 15], and enables continuity of carer [16]. It also provides for social support amongst group members, who, in our setting, may have become resident quite recently and/or may have migrated from abroad, where, for these and other reasons (e.g. financial constraints, limited English language ability), they may not have optimal existing support networks. Helping to address some of the main problems vulnerable and culturally and/or linguistically diverse women experience with standard, fragmented care, continuity of carer has been found to be beneficial, delivering enhanced communication and interpersonal rapport [17–19].

Furthermore, providing antenatal care within small groups promotes discussion and potentially, more effective learning for and amongst women, rather than solely relying on a health professional as the source of ‘expert advice’. It is also pertinent to note that many women living within the target areas do not currently have access to traditional antenatal education classes for various reasons. It is expected that this new approach will empower women, giving them more of ‘a voice’, enhancing informed decision making, and enabling them to tailor antenatal care more closely to their own needs. Significant benefits have been associated with such empowerment. If women feel that they have more autonomy and choice, this has been shown to increase their sense of control around birthing, and subsequently, this has the potential to increase their satisfaction with the birthing experience. Whether women experience birthing as a positive and affirming life event or as a traumatic, negative experience has the potential to affect their well-being and that of their children for the future life course [20–24]. The approach also encourages women to engage in more self-monitoring, with the aim of increasing knowledge and confidence; again, these factors have been shown to be significant in increasing the likelihood of a positive birth experience [25–28].

Although group antenatal care has been shown in other settings to be effective for improving women’s experiences of care and for improving other maternal as well as newborn health outcomes, these outcomes have not been formally assessed in the UK. A recent systematic review of group antenatal care concluded that more high-quality studies of its effectiveness are needed to establish whether positive findings are widely applicable [29]. These health improvements are in line with national and local aspirations for reducing inequalities and improving the health and well-being of women and children [23]. We are therefore proposing to evaluate robustly the effectiveness and cost-effectiveness of group-based antenatal care in enhancing women’s experience of antenatal care, increasing its relevance and value to women, and improving outcomes for mother and baby, particularly amongst women from ethnically, culturally and linguistically diverse and disadvantaged areas who are more likely to experience worse outcomes [30, 31]. This pilot trial is important to assess the best methods for conducting this proposed randomised controlled trial of this model of group antenatal care in the UK. Following best practice for pilot trials of complex interventions, pre-agreed progression criteria have been identified to inform the decision about whether to proceed, to proceed with amendments or not to proceed to a full trial (see Table 1) [32]. This paper relates to the pilot trial protocol version 2 (22.7.16) and adheres to the SPIRIT checklist reporting guidelines (Additional file 1).

**Table 1:** Progression criteria to full trial

	Green light	Amber light	Red light
Recruitment—numbers of available women in each catchment	Within each catchment area, 60+ pregnant women with appropriate due dates	Within each catchment area, 40–59 pregnant women with appropriate due dates	Within the catchment area, less than 40 pregnant women with appropriate due dates
Recruitment—percentage who consent to randomisation	More than 40% who are eligible consent to randomisation	20–40% who are eligible consent to randomisation	Less than 20% who are eligible agree to randomisation
Uptake of group care model	8 or more of the 12 randomised take up the groups	6–7 of the 12 randomised take up the group	Fewer than 6 of the 12 randomised take up the group
Retention in groups*	5 remain in the group for 6+ sessions	4–5 remain in the group for 6+ sessions	Fewer than 4 women remain in the group for 6+ sessions
Follow-up response rate—self-complete outcomes questionnaire(s)	75% or greater response to follow-up	40–74% response to follow-up	Less than 40% response to follow-up

*Allowing for preterm births and moving out of the NHS Trust usual catchment area

## Methods/design

### Pilot trial aims

This pilot trial aims to determine the optimum methods for testing the effectiveness of a bespoke model of group antenatal care, called ‘Pregnancy Circles’, in a UK NHS setting serving populations with high levels of social deprivation and cultural, linguistic and ethnic diversity. It will inform the protocol for a full randomised controlled trial of the Pregnancy Circles model. The pilot trial will be undertaken to include an assessment of:

1. Methods of recruitment, recruitment rates, and reasons for declining participation;
2. Retention in groups and reasons for drop out;
3. Data collection for outcome assessment;
4. Approaches to language support for intervention and research purposes.

An additional aim will be the further development and refinement of the Pregnancy Circles model of group antenatal care for culturally, linguistically, ethnically and socio-economically diverse communities. This will include determining the extent of the linguistic diversity that can be incorporated in a single Pregnancy Circle. The pilot trial will also aim to determine the suitability of our proposed primary outcome measure (spontaneous vaginal birth) and to develop a composite measure of a ‘healthy birth’ for the economic evaluation.

### Intervention

Following an extensive feasibility study, Pregnancy Circles are being implemented by a London NHS Trust as part of its service development. Initially, this was on a small-scale basis (four test groups in the feasibility work, followed by the three groups that form the focus of this pilot trial). Each Pregnancy Circle will consist of between 8 and 12 pregnant women who have estimated delivery dates within the same approximate 2- to 4-week period. The women who consent to participation in the study will be randomly assigned to one of two trial arms. One arm will receive standard antenatal care, whereas the other will receive all of their usual midwife-led antenatal care within a Pregnancy Circle. Any necessary appointments for consultant or specialist care will be carried out as per the usual care pathways outside of (and in addition to) the Circle.

Those women randomised to the group, antenatal care will start attending the ‘Pregnancy Circle’ for the first time at the routine midwife appointment (approximately 16 weeks of pregnancy) that follows their antenatal booking appointment (this usually takes place between 8 and 12 weeks of pregnancy). Subsequently, the women will continue to attend the Circle according to the normal antenatal care schedule. Any woman who chooses to discontinue her care in a group during pregnancy will transfer to the conventional care pathway. Any woman who does not attend the first Circle will be contacted by the facilitating midwives to ascertain the reasons for this. If non-attendance is due to pregnancy loss, they will be referred, by the facilitating midwives, to sources of ongoing support, such as their GP, as appropriate.

Each Pregnancy Circle group session will be facilitated by two midwives supplemented with bilingual health advocates or other support staff as appropriate. The same two midwives will facilitate all the sessions for a Pregnancy Circle, and each woman will have one of these midwives as their lead midwife. Women participating in the ‘Pregnancy Circle’ who are having their first child will receive the same number of antenatal appointments as women receiving standard care. Women having subsequent children will be offered the same number of antenatal appointments as women having their first child, therefore receiving two additional appointments to the standard model for women having subsequent children. All women attending the ‘Pregnancy Circle’ will receive standard postnatal care but will also be invited to a postnatal reunion session held approximately 1 month after the last antenatal appointment (40 weeks of pregnancy). A local health visitor will co-facilitate this reunion postnatal session with the midwives. Women in the control group will continue to have standard postnatal care and then standard health visitor care.

There will be a total of eight antenatal group sessions each of which will last for approximately 2 h. The first part of each session will involve ‘self-care activities’ (e.g. women will be taught how and encouraged to take an active part in their care by testing their own urine, taking their own/each other’s blood pressure and writing the results in their notes). Following these checks, the sessions will involve short one-to-one sessions with one of the midwife facilitators for individual health checks (e.g. abdominal palpation) whilst the rest of the group has a group discussion facilitated by the second midwife. Any concerns regarding a group member’s blood pressure or scan or test results, or any individual psychological or social issues, can be addressed during the individual health check or at the end of a session by a woman’s lead midwife, whilst the other midwife continues facilitating the group. The content of group discussions will be woman-led, supplemented as appropriate by the facilitating midwives to ensure that essential topics are covered, as per national and local guidelines. As with usual care, women will be referred to other specialist services for routine and additional appointments, blood tests and scans as appropriate. The postnatal session will use a similar approach and format, but with a focus on maternal postnatal well-being and the well-being of the baby and infant feeding support.

Midwife and health visitor facilitators will be documenting the appointment in the same way they usually would. During the first group session, the facilitating midwives will develop ground rules of confidentiality, asking the participants to respect each other’s privacy and confidentiality regarding what is shared within the group. The views of the group on how and when partners are involved in the sessions will be ascertained.

Pregnancy Circles model is a ‘bespoke’ model of antenatal care, designed to be flexible in order to allow the midwives and women to adapt it to suit local need—this may include the inclusion of external speakers, involving student midwives or other appropriate observers, or making adaptations for women with language or other needs.

### Study setting

The pilot trial will be carried out within the maternity services of an inner London acute NHS Trust. This Trust provides services primarily to residents of three London boroughs. One Pregnancy Circle (i.e. one group of women who have their antenatal appointments together) will be run within each of these three boroughs by midwives from the local service. The exact area within each borough in which the Pregnancy Circles will be run will largely be determined by practical issues, with decisions being made in consultation with service managers. The Circles will run in the usual working area of the midwives who facilitate them.

### Trial design

A UK-based external pilot randomised controlled trial will involve two parallel arms: one receiving a group model of antenatal care (Pregnancy Circles) and the other receiving usual care. It will not be possible for participants or maternity staff to be blinded to allocation. Researchers conducting process evaluation observations and interviews will also be unblinded. Data informatics staff supplying outcomes information from electronic records and those conducting the statistical analysis will be blinded to the intervention allocation.

#### Inclusion criteria

Eligibility will be assessed by a researcher or research midwife within the antenatal booking clinic. Those who will be eligible include:

- Women who are currently pregnant and registering (or registered) for antenatal care at the participating NHS Trust maternity service
- Primiparous and multiparous women
- All pregnant women, regardless of the risk category applied to their pregnancy (both ‘low’ and ‘high’ risk pregnancies included)
- Women who live within the working areas of the local midwife group facilitators and have an estimated delivery date that fits with those of a proposed group

The pilot will test groups with a limited diversity of languages spoken and groups with greater linguistic diversity (e.g. with one to three additional languages other than English). Thus, the recruitment for different Pregnancy Circles will have different inclusion criteria with respect to particular languages spoken.

#### Exclusion criteria

The following are the exclusion criteria:

- Non-pregnant women
- Women registered for antenatal care at other NHS Trusts
- Pregnant women who live outside the local target area
- Pregnant women whose estimated delivery dates do not fit with those of a proposed group
- Pregnant women who, at the time of antenatal booking, will likely not be able to participate at the proposed group start date and/or throughout the entire series
- Pregnant women who are under 16 years of age at the time of recruitment
- Pregnant women with a documented learning disability
- Pregnant women who speak a language that is not a target language for the group being recruited at the time of their potential recruitment

### Sample size

The study will aim to recruit 24 participants from each of the three maternity units within the participating NHS Trust making a combined total of 72 women. Half of the recruited women in each area (up to 12 women) will be randomised to take part in the ‘Pregnancy Circles’ and half to receive standard antenatal care. This sample will not be powered to show differences between the trial arms on outcomes. Rather, the sample size has been determined to allow us to test the assumptions used in the sample size calculations for a full trial. This sample size will allow recruitment to take place in the three geographical areas in the NHS Trust where the Pregnancy Circles will take place, to allow us to test recruitment rates and to assess whether this differs by area. It will also allow us to explore uptake and retention rates to see whether our assumptions about the loss to follow-up are appropriate.

### Recruitment of participants

Potential participants will be recruited from women attending their first midwife appointment to register with the NHS Trust maternity services (the ‘booking appointment’, which usually takes place when women are between 8 and 13 weeks pregnant). The research team will work with the antenatal booking clerks in each area to identify women who fit the inclusion criteria prior to the booking appointment. The participant information leaflet (PIL) about the REACH Pregnancy Circles study and an introductory letter will be included with the mailed booking appointment confirmation letter to these women.

Recruitment and consent will be carried out by REACH researchers and research midwives employed by the NHS Trust. Once attending their booking appointment, all women who fit the inclusion criteria will be approached by a researcher in the waiting room. An additional copy of the PIL will be provided. A verbal explanation of the Pregnancy Circles, the study and the concept of randomisation will also be given by the recruiter, and women will have the opportunity to ask questions, using interpreting services where appropriate.

Women who decline to participate in the pilot trial will be invited to take part in a brief (5-min) interview on their understanding of group antenatal care and their reasons for declining to participate. If they provide verbal consent to this 5-min interview, an anonymous proforma will be used to record their responses as well as basic demographic information and the date and venue of the clinic. These data will inform recruitment procedures for the potential future full trial. The interview will be with a researcher/research midwife at the time they decline to take part. It will be made very clear to women that their views will be used to inform efforts to recruit to future groups and that they are not being asked to justify or change their decision.

If a woman is unsure about whether she wishes to participate in the trial, she will be able to contact the researcher after the booking visit. She will be given a copy of the consent form and baseline questionnaire to take away to be completed at home if she decides to participate. Stamped addressed envelopes will also be provided. She will be asked if she is happy to give her telephone number/email address to the researcher so that a reminder contact can be made to clarify whether she is interested in participating. If a woman does not wish to give this contact information, she will have the option to telephone the research team using the number given on the PIL.

Women who choose to participate in the ‘Pregnancy Circles’ will be asked to sign a consent form and to fill in a self-completed baseline questionnaire. Language support, as described below, will be offered for the completion of both, if required. It will be explained in writing and verbally that they can withdraw at any time if they so wish.

### Language support

The study population is extremely diverse in terms of languages spoken. We will use the pilot trial to test out the feasibility and acceptability of different forms of language support. The principal forms of language support we plan to use in the trial are (1) bilingual health advocates employed by the NHS Trust^1^, (2) language line (a telephone interpreting service) and (3) bilingual advocates from an external agency.

We are particularly focussing on working in partnership with the health advocacy service within the NHS Trust which will be funded to provide services for the trial. This will include providing advocates to work alongside a researcher supporting recruitment and consent, telephone follow-up and questionnaire completion in clinic, community and home settings. Advocates will also work one-to-one with women in the Pregnancy Circles. We aim to provide training sessions about the research for the Trust’s health advocates as well as the staff who book advocates and attend meetings to facilitate joint working. We expect there to be occasions when an appropriate advocate is not available from the service, including when an eligible woman presents who speaks a language that is unusual for the area. In these instances, language line will be used for recruitment, and an external health advocate will be found to support further involvement.

### Randomisation

Participants will be allocated to Pregnancy Circles or usual care in a one-to-one ratio. Block randomisation will be carried out by a statistician in the Pragmatic Clinical Trials Unit (PCTU), independent of the trial. Randomisation will be stratified by the estimated delivery date and the location of the Pregnancy Circle. The research team will notify the woman verbally and in writing of her allocation and working with booking clinic staff will confirm either her next appointment date and time (for women allocated to the control group) or the full list of dates, times and venue for the Pregnancy Circles (intervention group).

### Pilot trial feasibility outcomes

**Fig. 1:**
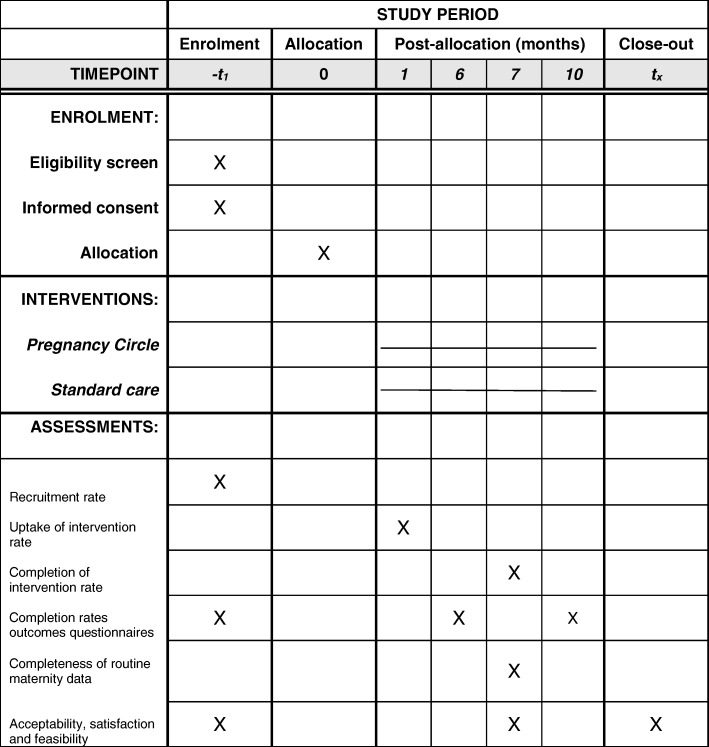
Schedule of enrolment, interventions and assessments. SPIRIT diagram for REACH Pregnancy Circles pilot study

Data will be collected on recruitment; uptake of, and retention in, the Pregnancy Circle groups; and outcomes data collection rates. Figure 1 highlights the assessments that will be completed by participants, as per the SPIRIT Statement [33].

The different rates achieved in the pilot will enable estimates to be made for a full trial. Additionally, they will inform decisions about changes that need to be implemented and the appropriateness of progression to such a trial; this process will be undertaken in conjunction with the Programme Steering Committee (see Table 1). ‘Red light’ rates will halt the progression, unless suitable solutions can be implemented.

(i) *Recruitment figures* will be kept for each antenatal booking clinic session attended by the research midwives via a proforma, regarding numbers of women eligible, number approached, number who declined and number who consented. No identifying information will be recorded. Approaches used for language support will be recorded.
(ii) *Uptake and retention to the intervention* will be derived from records kept by the midwives who are facilitating the Pregnancy Circles, who will record attendance at each group using a paper proforma, which they will pass on to the research team. The research team will transfer this information to a password-protected electronic database and replace names with study IDs. The paper proformas will then be destroyed. Midwives will have a protocol to follow for women who choose not to participate in the Circle, at any point through the intervention, to ensure that all women receive antenatal care.
(iii) *Questionnaire completion rates will be used to assess* the potential burden of data collection for participants. We will explore completion rates at each of the two follow-up points and whether each measure within the questionnaire is completed in a way that provides useable data. We will explore the questionnaire completion rates for women who required a bilingual advocate, to provide further information about the appropriateness of our research tools. Additionally, this will be assessed through open questions during one-to-one interviews with participants (see the “Process data—views about trial methods and the intervention” section below). More detail is provided on the questionnaires in the “Participant outcomes data collection” section below.

#### Process data—views about trial methods and the intervention

Process data will also be collected from participants and key stakeholders to assess the feasibility of the proposed methods for the full trial of ‘Pregnancy Circles’. The aim of this data collection will be to help explain the reasons behind any challenges with recruitment, uptake, retention and outcomes data collection and to provide solutions for improvement. This data will be collected through process questions added at the end of the outcomes questionnaires (see Table 2) and one-to-one interviews with a small sample of women and staff.

**Table 2:** Data measures for Pregnancy Circles pilot

Baseline measures (12 weeks pregnant)	Outcomes questionnaire 1 (35 weeks pregnant)	Postpartum maternity records audit	Outcomes questionnaire 2 (4 months postpartum)
Social support (Duke Social Support Scale) Self-efficacy (Pearlin Mastery Scale) Prenatal stress (Revised Prenatal Distress Questionnaire) Health service usage maternal self-report of their use over the previous 4 months of a variety of primary health services (GP, health visitor, social work and hospital doctor), A&E services, antenatal admissions. Demographic questions • Age • Ethnicity • Language • Parity • Education • Tenancy	Women’s satisfaction with care (questions from the Care Quality Commission’s Maternity Survey) Social support (Duke Social Support Scale) Self-efficacy (Pearlin Mastery Scale) Involvement in decisions about care (Picker Institute’s Experience with Maternity Services questionnaire) Prenatal stress (Revised Prenatal Distress Questionnaire) Health service usage maternal self-report of their use over the previous 4 months of a variety of primary health services (GP, health visitor, social work and hospital doctor), A&E services, antenatal admissions. Attendance at antenatal education classes (NHS; other) Process questions • Randomisation feelings *For intervention:* • Attendance • Reasons for non-attendance • Perceptions of the group experience	Spontaneous vaginal birth (*primary outcome*) Attendance at ANC (number of regular appointments attended) Caesarean delivery (planned, emergency, none) Epidural/spinal analgesia use in labour Infant birthweight, defined as low if less than 2500 g Gestational age at delivery, dichotomized as term or preterm (less than 37 weeks) Breastfeeding initiation	Women’s satisfaction with care (questions from the Care Quality Commission’s Maternity Survey) Social support (Duke Social Support Scale) Self-efficacy (Pearlin Mastery Scale) Involvement in decisions about care (Picker Institute’s Experience with Maternity Services questionnaire) Breastfeeding continuation and exclusivity Health service usage maternal self-report of their use over the previous 4 months for themselves and their baby of a variety of primary health services (GP, health visitor, social work and hospital doctor), A&E services, late antenatal admissions, and uptake of infant immunisations at 2 and 3 months). Postnatal depression (Postpartum Depression Screening Scale) Process questions • Perceptions of the impact of ANC on birth/postpartum • Views of trial procedures

The process evaluation will include the following components:

- *Five-minute interview and anonymous proforma for women who decline to take part in the study*, as described in the ‘Recruitment of patients’ section above.
- *Interviews with Pregnancy Circle participants*

At the final antenatal group session, participants in the Pregnancy Circles will be offered the opportunity to have a one-to-one semi-structured interview after their baby has been born. This will last 30–60 min and will be conducted either face-to-face or via the telephone according to participant preference. A purposeful sample of approximately 20 participants will be interviewed, at a time (probably around 6 weeks postnatal) and location of their choice. Women may choose to have their partners and/or children with them during the interview. Women who have suffered an adverse neonatal outcome (neonatal death, admission to the neonatal unit) will not be interviewed unless they specifically request it. The facilitating midwives will confirm whether women who had previously consented to the interview can be contacted postnatally. The purpose of the interviews will be to explore their experiences and satisfaction with this model of antenatal care and their perceptions of its effects, as well as their views on recruitment, randomisation and data collection methods. The views and experiences of women who received language support will also be sought.

c) *Interviews with women who leave the ‘Pregnancy Circles’*

Women who discontinue with a group during pregnancy will be invited to participate in an interview (up to 30 min, in person or by telephone depending on preference). Women who suffered pregnancy loss will not be approached for these interviews. The purpose of the interviews will be to explore the reasons the woman has decided to leave the group, and if relevant, explore how the group failed to match the women’s expectations, including the content, convenience and conduct of the sessions. The interviews will elicit suggestions for future groups and explore women’s views about randomisation to this kind of care.

d) *Interviews with facilitating midwives and other staff involved with ‘Pregnancy Circles’*

In order to provide context to data collected from women, a purposeful sample of midwives and other relevant staff at the participating NHS Trust will be offered the opportunity to take part in a brief (up to 30 min) interview about their perceptions of the recruitment and randomisation procedures, the data collection methods (e.g. timing) and issues relating to delivery of and retention in the Pregnancy Circles. Interviews with midwives will include questions pertaining to potential contamination of usual care by the introduction of Pregnancy Circles care.

Information about the process evaluation participants (name, contact details, demographic details) will be stored on a participant information database on the secure server at City University London. Study ID numbers will be used on collected data to ensure anonymity. Signed consent forms for process data collection will be stored separately in a locked filing cabinet in the office of the research team at UEL, which requires an access code to enter. The interviews will be transcribed verbatim, and the confidentiality of personal data will be ensured through the use of anonymisation and pseudonymisation techniques. All audio recordings and transcripts will be stored securely in a locked filing cabinet, in a locked office, that only members of the research team will have access to. Transcribers will be bound by a confidentiality agreement.

All qualitative interview data will be entered into the data analysis package NVivo, which will be used to manage and code data. The data will be subjected to thematic framework analysis. Codes will be applied line by line to transcripts, which will identify key themes and how these inter-relate in order to develop an analytical framework.

### Participant outcomes data collection

Participant outcomes data in a prospective full trial will be collected via two routes: questionnaires completed by the participants and routine maternity service data collected from the Trust. The task in the pilot, as explained, is to assess the completion rates and also to determine the optimum processes for collecting useable outcomes data.

#### Outcomes questionnaires general overview

The questionnaires will be administered as online and hard copies, with participants able to select their preferred mode. The research team will use a database to track the sending out and return of hard copies. To facilitate online surveys, an electronic participant-recorded outcome tool, provided by the PCTU, will be used. This will have the facility to email participants a link to questionnaires. All women will be offered a £10 voucher for each of the three questionnaires they complete. These will be handed to women who complete their questionnaire in the presence of a researcher and posted to women who submit their questionnaire without a researcher being present.

The questionnaires will be identified only with study ID number. The hard copies of the questionnaires will be stored in a locked filing cabinet in a pin code accessible office for the duration of the study. Data will be entered onto a database developed and maintained by the PCTU. For hard copy questionnaires, research team staff will do full double data entry for the primary outcome and double data entry on a 10% sample for other outcomes. Depending on identified error rates, the proportion of data double entered may be increased.

The participants’ questionnaires will be completed at three time points: baseline (8–12 weeks pregnant), first follow-up questionnaire (35 weeks pregnant) and second follow-up questionnaire (approximately 4 months after the baby is born). The baseline questionnaire will include demographic questions, a limited number of outcome measures and some questions relating to service preferences. The follow-up questionnaires will include outcome measures and some process questions relating to randomisation, attendance at antenatal care and preferences for providing outcomes information, see Table 2 for details on the questionnaires.

#### Baseline questionnaire

The baseline questionnaire will be provided by the researcher, as a hard copy or electronically on a tablet computer, for immediate completion following recruitment in the antenatal clinic. Completion will be prior to trial arm allocation being revealed to the women in order that knowledge of the type of care they will receive does not influence their responses. Women who cannot complete immediately, or want time to consider participation in the trial, will be given a hard copy (and an addressed/postage paid envelope) to post back or they can be provided with a link to the online survey to complete at home. These women will be informed of their trial arm status once the questionnaire has been received. If questionnaires that are taken away are not returned after 2 weeks, a telephone call will be made to offer completion over the phone with a researcher. For those women who do not speak English or who have limited literacy, the researchers will offer to make home/community visits to administer the questionnaires accompanied by a bilingual health advocate where required.

Feedback from our patient and public involvement (PPI) representatives on the draft baseline questionnaire suggested that this should be as short and simple as possible to be acceptable to the diverse community in the study. The validated measures within the questionnaire were perceived as particularly challenging and off-putting to potential participants. Two versions of the questionnaire will therefore be used to test acceptability—one, a shortened version of the other. A randomly allocated schedule of distribution of the two versions will be developed by an independent statistician. Approximately half the recruited women will be given the longer version and half the shorter, via sealed and numbered envelopes. Recruiters will be unaware of the allocation. Acceptability to participants of the different versions will be assessed by relative completion rates. The statistical implications of not having certain data at baseline will also be reviewed.

#### Follow-up outcomes questionnaires

For the intervention group, the first follow-up questionnaire will be provided at the completion of the Pregnancy Circle session when women are 35 weeks pregnant. A researcher will provide a hard copy or online version (on a computer tablet) depending on a woman’s preference. Interpreters will be at the group, where appropriate, and will assist with completion. Anyone who would prefer to complete the questionnaire at home, for whatever reason, will be supported to do so. For the control group, the questionnaire for the first follow-up will be completed, with the support of a researcher, in the antenatal clinic at a woman’s 35-week routine appointment. Where this is not possible for any individual woman, the questionnaire will be sent out for completion at home.

For women in both arms of the trial, the second follow-up questionnaire will be completed at approximately 4 months postnatal. Depending on earlier specified preferences, women will be sent hard copies to home addresses with a self-addressed envelope (SAE), and/or women will be emailed a link to an online version.

The research team will check with antenatal clinic staff, prior to contacting women about follow-up questionnaires, to ensure that there are no reasons (for example the loss of a pregnancy) why a woman should not be approached. The same pattern of reminders for non-response to the follow-up questionnaires will be followed as used for the baseline questionnaire (see above). Similarly, visits will be offered with a bilingual health advocate where there are language requirements. Any woman who requests withdrawal from the intervention following allocation will be asked if she is prepared to continue to provide data for the study. If she is, she will be given outcomes questionnaires as above.

#### Routine maternity services data

As Table 2 indicates, some participant outcomes data (including the primary outcome data) will be assessed through patient records, rather than from self-completed questionnaires. Specific permission for use of patient records is part of the study consent form. The pilot trial will use two routes to access these data: (1) through electronic medical records and (2) through an audit of paper maternity notes. The research team will check reliability and data quality of the two versions of medical records and will thus determine the best route for accessing the required outcomes data in a full trial. The team’s previous work undertaken via a Programme Development Grant suggested that some outcomes data on attendance for antenatal care was complete in maternity notes but incomplete in the electronic record [34].

For the data extraction from electronic patient records, the research team will develop a proforma for the Trust Informatics team (the team responsible for managing electronic data). We will supply them with a list of participants’ hospital numbers and study IDs, so no names will be required. A standard operating procedure (SOP) will be generated, in conjunction with the data management team at the PCTU, for the secure transfer of data from the Trust Informatics departments to the PCTU.

For the paper maternity notes, a research team member who is blinded to the allocation to study group will conduct an audit by extracting data manually from the participants’ paper records within the hospital setting. A proforma will be used that will anonymise the information collected, using study ID numbers. The data will be entered onto an electronic database and securely transferred to the PCTU using the SOP developed by the PCTU data management team.

#### Method of analysis

Participant outcomes data will be analysed for completeness and for usability in a full trial. Outcomes will be summarised in each arm using mean and standard deviation for continuous data, number and percentages for categorical data.

#### Data handling

The PCTU data managers will co-ordinate the management process for the outcomes data. Electronic systems will be put in place that automatically manages the process of sending out vouchers and reminders for questionnaires. It will also involve supporting the integration of the outcomes data from participant questionnaires, electronic maternity records and maternity audit data. A data management plan will document the processes required to enable integration of the datasets in a manner that maintains standardisation of data and blinding of the statisticians. All databases and analysis files will be stored on a secure server and accessed via a secure network. Access is restricted to authorised personnel only and via secure, password-controlled, role-based access.

### Economic evaluation

In the full trial, we plan to calculate the cost-effectiveness of Pregnancy Circles compared to control from conception until 4 months postpartum. NICE recommend that cost-effectiveness is calculated as the cost per quality-adjusted life year (QALY) gained [35]. This methodology presents significant challenges for this study. Group antenatal care has potential health implications and costs for both the mother and infant, in particular as a key aim of group antenatal care is better engagement of women with maternity and other health care services. This could potentially increase some service costs in the short term, but also improve the health and well-being of the infant and mother in the immediate period, and also in the medium and longer terms. Increasing the proportion of infants vaccinated is the most straightforward example of improved engagement with health care services. Although measuring QALYs for the mother is possible, when and how to start measuring QALYs from the perspective of the infant is controversial and methodologically challenging.

In the pilot trial, the economic evaluation will explore alternative utility measures via the participant questionnaires. Additionally, we will involve women in defining what a ‘healthy birth’ is, so that we will be able to calculate the incremental cost per additional healthy birth for group antenatal care compared to control, using this composite measure. In the early months of the pilot, we will work in consultation with our lay co-investigators and with the local Maternity Services Liaison Committees, to determine whether the suggested questions to be used in the follow-up questionnaires to capture women’s satisfaction with antenatal care are sufficient, or whether additional ones need to be included. Responses to antenatal care satisfaction questions plus relevant outcomes data will be included in the composite. Initially, we will conceptualise a ‘healthy birth’ as occurring if women:

1. Were largely satisfied with their care during pregnancy
2. Felt involved in decision-making about their care
3. Had low antenatal distress
4. Did not suffer postnatal depression (within the follow-up period)
5. Perceived that they had good levels of information and professional support (during pregnancy, labour and delivery)
6. Had a baby with gestational age at delivery, ≥ 37 weeks.
7. Did not have a caesarean
8. Had a baby with a birthweight > 2500 g
9. Felt their care was responsive to cultural, religious/spiritual and linguistic needs (i.e. individualised, culturally sensitive care).

We will use the data collected during the pilot to assess the validity of this composite measure and to develop algorithms for how births might be classified on a continuum from ‘healthy’ to ‘unhealthy’.

Our assumption for the full trial is that costs will include the cost per pregnant woman of group antenatal care as well as mother and infant primary and secondary care costs from conception until 4 months postpartum. As such, in this pilot trial, use of primary and secondary care will be collected via additional questions on the participants’ baseline and follow-up questionnaires. Unit costs will be obtained from published national sources. Confidence intervals and cost-effectiveness acceptability curves will be calculated using bootstrapping (sampling with replacement). The perspective we will adopt for the economic evaluation will be health and social care costs. We will validate our resource use data as part of the pilot study by checking resource use reported by women in a sample of questionnaires against medical records. For the sensitivity analysis, we will use non-parametric bootstrap to construct confidence intervals for trial data: one-, two- and multi-way deterministic analyses for any assumptions made; if a decision model is constructed, we will do a full probabilistic sensitivity analysis. Trial missing and censored data will be handled the same way in the economic evaluation as for the statistical analysis.

### Monitoring and auditing

The pilot trial will be overseen by a steering committee. This group will meet at least once face-to-face during the 15 months of the pilot trial and then continue to meet yearly if the main trial goes ahead. This committee will be responsible for overseeing the pilot and the main trial, ensuring scientific quality and clinical relevance, and adherence to ethics and research governance. All key collaborators on the pilot trial will attend this committee, as well as a range of experts who are not directly involved in the pilot, including a chair with relevant expertise, a statistician, an economist and a PPI member.

The PCTU quality assurance (QA) manager will conduct a study risk assessment in collaboration with the CI. Based on the risk assessment, an appropriate study monitoring and auditing plan will be produced according to PCTU SOPs. This monitoring plan will be authorised by the sponsor before implementation. The PCTU QA manager and the sponsor will agree on any changes to the monitoring plan. The CI will ensure that safety monitoring and reporting is conducted in accordance with the sponsor’s requirements. This will include appropriate processes for management of adverse events.

### Patient and public involvement

There has been extensive prior public consultation in the study area, including interviews and discussion and stakeholder group meetings. Additional to the feasibility work already described involving local mothers/women and the qualitative work with women conducted as part of the programme development grant [36], the REACH Pregnancy programme has two lay co-investigators who have contributed to the development of the protocol, the participant information sheets, recruitment methods and data collection instruments, as did members of a patient and public involvement group run by a local women’s health research network. The City University Research ‘Advisory Group for Maternal and Child Health Research’ have also been consulted about potential methods of recruitment. The lay co-investigators and the patient and public advisory group will also be invited to contribute to data analysis, decisions relating to dissemination products and processes, review lessons learnt and implications for a future trial. The REACH Pregnancy Programme is also a standard item on the agenda of three local Maternity Services Liaison Committees’ (MSLCs). Members of MSLCs include NHS maternity staff and local representatives of maternity services users.

### Dissemination

The findings of this pilot trial will be presented at national and international conferences (e.g. Royal Colleges of Midwives annual conference, the International Confederation of Midwives Congress and relevant national public health conferences) and published in peer-reviewed academic journals. Additionally, findings will be made available in accessible formats in newsletters and on the study website, as well as in professional and practitioner journals. The findings will also be reported as briefing papers to healthcare commissioners and managers and to service users via Maternity Service Liaison Committees. We will use links with the Reproductive and Childbirth topic network to further disseminate throughout the NHS.

## Discussion

This pilot trial will determine whether a full multi-centre RCT of Pregnancy Circles can be achieved in an NHS setting serving populations with high levels of social deprivation and cultural, linguistic and ethnic diversity and, if so, the optimum methods for doing so. Our pre-set progression criteria should be invaluable in supporting key decisions. It will also help to inform the protocol for a future full trial.

Specific design and methodological decisions that the pilot trial will also assist with include (a) whether spontaneous vaginal birth is the most appropriate primary outcome measure, and if not, what is a better alternative; (b) the components of a measure of ‘healthy birth’ for economic evaluation purposes; and (c) the best source (either electronic or paper records) of routine maternity data. It will provide invaluable data about the involvement of women who do not speak English, in terms of best methods for recruitment and follow-up, the appropriateness of using scales validated in English with them (via bilingual advocates) and for their involvement in group antenatal care.

If progression to a full trial is supported, using our pre-determined criteria, the pilot will provide authoritative, high-quality evidence to inform the design and conduct of a trial in this important area where it appears significant potential exists for improvements in health care and women’s experiences.

## Additional file

Additional file 1: Spirit Checklist for Manuscript of Protocol paper: testing the effectiveness of REACH Pregnancy Circles group antenatal care: protocol for a randomised controlled pilot trial Spirit checklist for this protocol. (PDF 119 kb)

## Abbreviations

A & E: Accident and emergency
CI: Chief investigator
HV: Health visitor
ID: Identification
NHS: National Health Service
PCTU: Pragmatic Clinical Trials Unit
PIL: Participant information leaflet
PPI: Public and patient involvement
QALY: Quality-adjusted life year
REACH: Research for Equitable Antenatal Care and Health
REC: Research Ethics Committee
SAE: Self-addressed envelope
SOP: Standard operating procedure
UK: United Kingdom

## Footnotes

1 This is the title given by this health service to those employed to provide language interpreting services.
